# Additive effect of *MTHFR *and *GRIN1 *genetic polymorphisms on the risk of schizophrenia

**Published:** 2015-01

**Authors:** Ali Mohammad Foroughmand, Hamid Galehdari, Atefeh Pooryasin, Tahereh Ajam, Seyed Reza Kazemi-Nezhad

**Affiliations:** Department of Genetics, College of Sciences, Shahid Chamran University, Ahvaz 61357-44337, Iran

**Keywords:** Homocysteine, *GRIN1*, *MTHFR*, NMDA, Schizophrenia

## Abstract

Schizophrenia is a complex disorder with polygenic inheritance. The *MTHFR *gene (OMIM: 607093) plays an important role in the folate metabolism. It has been suggested that C677T (rs1801133) and A1298C (rs1801131) genetic polymorphisms in the *MTHFR *gene lead to the decreased activity of the methylenetetrahydrofolate reductase enzyme which may have significant effect on developing schizophrenia. We used a case-control study to establish the possible association between the C677T and the A1298C polymorphisms and susceptibility to schizophrenia in an Iranian population. The genotypes of the polymorphisms were determined using PCR-RFLP. The data were analyzed by logistic regression model. Data analysis revealed that the combination genotypes of 677CT/1298AA, 677CC/1298CC, 677TT/1298AA, 677CT/1298AC and 677CT/1298CC increase the risk of schizophrenia. In order to evaluate the effect of combined genotypes of the three mentioned polymorphic loci, the frequencies of the compound genotypes were compared between control and patient groups (Table 4). Base on the results, the existence of >4 risk factors showed about 32-fold increased risk for schizophrenia (OR=32.3, 95% CI: 5.52-188, P=<0.001).

## INTRODUCTION

Schizophrenia is a complex psychotic disorder with a multiple gene inheritance, which affects approximately 1% of the general population worldwide [1]. In the past few years, several lines of evidence are suggesting that altered folate metabolism may increase vulnerability to schizophrenia [2].Folate is an important B vitamin that plays a pivotal role in remethylation of homocysteine to methionine, which is essential for DNA-synthesis, DNA-repair and DNA-imprinting processes [3]. Reduction of 5, 10-methylenetetrahydrofolate into 5- methyltetrahydrofolate, the predominant circulatory form of folate is catalyzed by the 5,10-methylenetetrahydrofolate reductase (MTHFR), the regulating key enzyme for availability of active folate at the expense of elevated homocysteine levels [4]. The 5- methyltetrahydrofolate donates a methyl group to homocysteine in generation of S- adenosylmethionine (SAM), a major source of methyl groups in the brain [5].

The *MTHFR *(OMIM: 607093) gene is located at 1p36.3. Positive linkage of 1p36 with schizophrenia has been reported [6]. Two common polymorphisms in *MTHFR *gene, C677T (rs1801133) in exon 4 and an A1298C (rs1801131) in exon 7 are functional and result in diminished enzyme activity [7, 8]. Homozygote variants have 30 and 60 percent enzyme activity in comparison with homozygotes for the wild-types, for the C677T and A1298C polymorphisms, respectively [9, 10]. MTHFR deficiency has been associated with reduction in folate acid metabolism and hyperhomocysteinemia [11, 12]. The involvement of *MTHFR *in schizophrenia has been confirmed by the observation of clinical improvement in this psychiatric illness with folate [13]. Also, some authors have reported hyperhomocysteinemia in their schizophrenic patients [14-16]. Higher homocysteine concentrations were especially found in plasma of subjects with the *MTHFR *677TT genotype with low plasma folate concentrations compared to subjects with the normal genotype [7, 17]. Several studies have been reported association between *MTHFR *polymorphisms and schizophrenia, but the results are not consistent [18-27].

The pathogenic mechanisms by which the hyperhomocysteinemia causes diseases are not fully understood, but there is an evidence that homocysteine disrupts the neurodevelopment by acting as an antagonist of the N-methyl-D-aspartate (NMDA) receptor glycine co-agonist site [28]. Furthermore, the NMDA receptor, a member of the family of ionotropic glutamate receptors, functions as a glutamate-gated cation channel [29]. These channels are implicated in both neural cell survival and neurotoxicity, and also have important role in development, synaptic plasticity and long term potentiating [30]. This receptor is a heterodimer consisting of the NR1 and NR2 (NR2A-D) subunits [31]. It has proposed that the hypofunction of NMDA receptors might be involved in the pathophysiology of schizophrenia [32]. The glutamate hypothesis of schizophrenia is based largely on the observation that NMDA receptor antagonists such as PCP, MK-801, and ketamine, produce both the positive and negative symptoms associated with this disorder. Furthermore, clinical trials have shown that treating patients with drugs that promote NMDA receptor function, such as D-serine, improves cognitive deficits associated with schizophrenia. In addition several lines of evidence have implicated that the N-methyl D-aspartate 1 (*GRIN1*; OMIM:138249) plays a fundamental role in many brain functions and its involvement in the pathogenesis of schizophrenia has been widely investigated [33]. Recently we found a positive association between G1001C polymorphism (rs11146020) in the promoter of *GRIN1 *and developing of schizophrenia [33].

In the present study, association between *MTHFR *polymorphisms and susceptibility to schizophrenia was investigated. According to possible effect of both *MTHFR *polymorphisms and the G1001C variant of *GRIN1 *on NMDA receptor activity, we also investigated combination of these polymorphisms as a potential risk factor for developing schizophrenia.

## MATERIALS AND METHODS

**Subjects: **Two hundred (male 117, female 83) unrelated patients with a mean age of 43.3 (SD = 11.3) were recruited from hospitals in south and southwest Iran. The control group consisted of 200 healthy blood donors (male 117, female 83) with a mean age of 39.4 (SD = 11.1), which were matched for gender and ethnicity to the patient. Informed consent was obtained from all participants.

**Genotyping assay: **Genomic DNA was extracted from leucocytes using standard salting out method. The C677T and A1298C polymorphisms in *MTHFR *gene were determined by PCR-RFLP using *Hinf*I and *Mbo*II restriction enzymes, respectively. To confirm the results, a random selection of 20% of all samples was retested. There were no discrepancies in replicating test. The evaluation of *GRIN1 *polymorphism has been explained in details and reported before [33].

**Statistical analysis: **Comparison of genotypes between gender groups was done by chi square-test. The association between genotypes and development of schizophrenia was examined using odds ratios (ORs) and 95% confidence intervals (CIs). Statistical significance was considered at P< 0.05.

## RESULTS

The study of socio-demographic features of this case-control study identified that subjects in the case group were significantly lower than those in the control group in terms of marital status and educational level (Table 1).

Control and patient groups were initially classified according to their gender. Considering that there was no statistical difference between their genotypic frequencies (P>0.05), the samples were pooled. Detailed results of genotyping assay for C677T polymorphism are shown in Table 2. Genotype distributions were in Hardy-Weinberg equilibrium for both C677T and A1298C polymorphisms in patient and control groups.

In our samples, neither heterozygosity (OR=1.40, 95 % CI: 0.92-2.14, P=0.115) nor homozygosity (OR=1.82, 95% CI: 0.86-3.83, P=0.116) of the T allele was associated with the increasing risk of schizophrenia (Table 2). There was significant linear trend in associated risk with zero, one, and two T alleles (P=0.044). For the A1298C polymorphism, a significant association between the CC genotype and schizophrenia risk was identified (OR=2.04, 95% CI: 1.14- 3.66, P=0.016). There was a significant linear trend in associated risk with zero, one, and two C alleles (P=0.039).

**Table 1:** The socio-demographic characteristics of the case and control samples

**Variables**	**Controls (n=200)**	**Patients (n=200)**
Age (mean±SD) Age of onset (mean±SD)	39.4±1 -	43.3±11.3 22.0± 9.0
**Educational level (%)**		
Illiteracy	3 (1.5)	29 (14.72)
Primary school	13 (6.5)	119 (59.5)
High school	145 (72.5)	42 (22.1)
College	39 (19.5)	7 (3.68)
**Marital status (%)**		
Single	43 (21.5)	139 (69.5)
Married	156 (78)	35 (17.5)
Divorced	1 (0.5)	16 (8)
Uncertain	-	10 (5)

**Table 2:** Association between C677T and A1298C polymorphisms of *MTHFR* and schizophrenia risk

**Genotypes**	**Controls**	**Patients**	**OR**	**95 % CI**	**P-Value**
**C677T polymorphism**					
**CC**	123	104	1.0	-	-
**CT**	64	76	1.40	0.92-2.14	0.115
**TT**	13	20	1.82	0.86-3.83	0.116
**A1298A polymorphism**					
**AA**	65	60	1.0	-	-
**AC**	108	89	0.89	0.57-1.39	0.621
**CC**	27	51	2.04	1.14-3.66	0.016

**Table 3:** Comparison of the combination of C677T and A1298C genotypes of *MTHFR* within control and patient groups

	**A1298C**	**Controls**	**Patients**	**OR**	**95% CI**	**P-value**
CC	AA	35	16	1.0	-	-
CC	AC	67	49	1.60	0.79-3.21	0.186
CT	AA	19	26	2.99	1.29-6.91	0.010
CC	CC	21	39	4.06	1.83-8.99	0.001
TT	AA	11	18	3.58	1.37-9.30	0.009
CT	AC	40	39	2.13	1.02-4.46	0.044
CT	CC	5	11	4.81	1.43-16.1	0.011
TT	AC	1	1	2.18	0.12-37.2	0.588
TT	CC	1	1	2.18	0.12-37.2	0.588
						

**Table 4:** Association between putative high risk alleles of *MTHFR *and *GRIN1* polymorphisms and risk of schizophrenia

**Number of putative high risk alleles**	**Controls**	**Patients**	**OR**	**95% CI**	**P-Value**
**0**	19	2	1.0	-	-
**1**	48	29	5.74	1.24-26.4	0.025
**2**	81	80	9.38	2.11-41.6	0.003
**3**	47	72	14.5	3.23-65.3	<0.001
**>4**	5	17	32.3	5.52-188	<0.001

We next studied the joint effect of the two polymorphisms (Table 3). The combination genotypes of 677CT/1298AA, 677CC/1298CC, 677TT/1298AA, 677CT/1298AC and 677CT/1298CC increase the risk of schizophrenia.

In order to evaluate the effect of combined genotypes of the three mentioned polymorphic loci, the frequencies of the compound genotypes were compared between control and patient groups (Table 4). Base on the results, the existence of >4 risk factors showed about 32-fold increased risk for schizophrenia (OR=32.3, 95% CI: 5.52-188, P=<0.001).

## DISCUSSION

In the present investigation, we did not find any significant association between the C677T polymorphism and schizophrenia. Our result is in accordance with some other investigations [19, 20, 22, 23, 34]. But, in contrast with our result, some case-control and family-based studies supported the relationship between T677T genotype and the rising of the schizophrenia risk [18, 21, 24, 25, 27].

The second polymorphism in the *MTHFR *gene, the A1298C, reduces enzyme activity for about 30-40% [10, 35] and the possible effect of this polymorphism in schizophrenia was examined in some investigations [24, 26]. In this study, we also found the 1298CC genotype as a risk factor in schizophrenia. In contrast, other researchers could not find this relationship [22, 23]. The A1298C polymorphism has been shown being associated with hyperhomocysteinemia [9, 35] while homocysteine has implicated as a risk factor in schizophrenia [14, 15, 36, 37].

Previous studies postulated a combined association between the A1298C polymorphism and the C677T variant with MTHFR activity and homocysteine levels [38]. Although we did not find any effect of the C667T polymorphism as a risk factor in schizophrenia. But significant association was revealed between schizophrenia and combinations of the A1298C and the C677T polymorphisms (Table 3). The recent meta-analysis reinforced the effect of the *MTHFR *on developing schizophrenia [39].

Low contents of the SAM in human tissues could result from deﬁciency in the *de novo *biosynthesis of the methyl group using folate-dependent one-carbon pathway [7, 40]. The MTHFR enzyme plays a central role in folate metabolism [5]. The C677T and A1298C polymorphisms have been associated with up to a 70% reduction in folate acid metabolism [11, 12]. On the other hand, schizophrenia is considered to be a neuro- developmental disorder [41] which epigenetic mechanisms such as DNA methylation might be important in its etiology [42, 43]. DNA methylation is a critical epigenetic modifier of the genome that controls many biologic processes, including embryonic development, X-chromosome inactivation, imprinting, and gene expression. The link between folate, folate metabolism, and DNA methylation therefore provides a plausible biologic mechanism for the observed association between the MTHFR gene and schizophrenia [44]. Reduced MTHFR activity has been found in brain tissue of schizophrenia patients [45]. In the brain, SAM is directly involved in the synthesis and metabolism of dopamine, epinephrine and serotonin, which as neurotransmitters play a crucial role in the pathogenesis of neurological disease [46].

Hyperhomocysteinemia can result from decreased MTHFR enzyme activity, owing to genetic polymorphisms [47]. Regland et al., demonstrated abnormally elevated homocysteine levels in 45% of schizophrenia patients [14]. It have been reported that homocysteine levels are elevated in both chronic and newly admitted schizophrenic patients [48, 49]. Hyperhomocysteinemia could lead to the mild cognitive impairments [50]. The homocysteine may contribute in developing schizophrenia by increasing oxidative stress processes [51, 52] and inducing DNA strand breakage and apoptosis [53-55]. Note worthily, the homocysteine was exerting an NMDA antagonist effect [56].

To our knowledge, the present study is the first to evaluate this combination effect. Interestingly, we found a significant association between the coexistence of these polymorphisms and increase the risk of schizophrenia.

## Notes

**Acknowledgements: **We thank Research Deputy of Shahid Chamran University supporting this work and all of the participants have been making this research possible.
